# Supplementary material for: Spontaneous Depolarization-Induced Action Potentials of ON-Starburst Amacrine Cells during Cholinergic and Glutamatergic Retinal Waves
Source: Cells. 2020 Dec 1;9(12):2574. doi: 10.3390/cells9122574 (PMC7759856; doi:10.3390/cells9122574)
Supplement: Supplementary file 1 [file cells-09-02574-s001.pdf]

Spontaneous depolarization-induced action potentials of ON-starburst amacrine cells during cholinergic and glutamatergic retinal waves

Rong-Shan Yan[1,2], Xiong-Li Yang[1,3], Yong-Mei Zhong[1,3*] and Dao-Qi Zhang[2*]

[1]Institutes of Brain Science, Fudan University, Shanghai, China
[2]Eye Research Institute, Oakland University, Rochester, MI
[3]State Key Laboratory of Medical Neurobiology and MOE Frontiers Center for Brain Science, Fudan University, Shanghai, China

***** Correspondence: ymzhong@fudan.edu.cn (Y-M.Z.); zhang@oakland.edu (D-Q.Z.)

Supplementary Figures

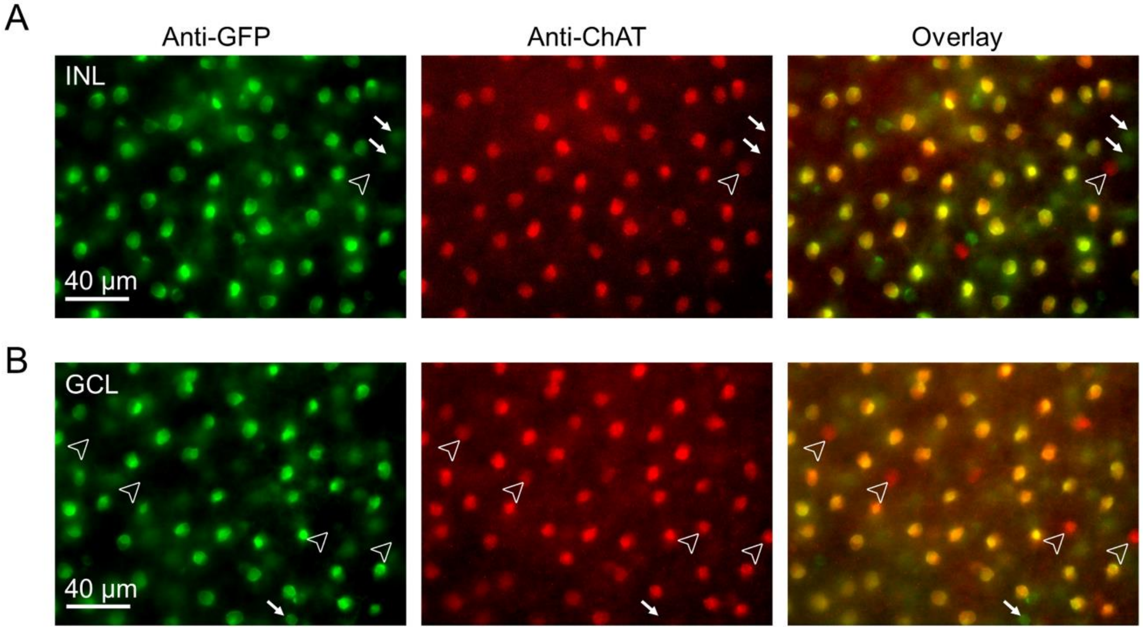

Supplementary Figure 1. Starburst amacrine cells are genetically labeled in ChAT-Cre/GCaMP6f mice. Retinas were isolated from the eyes of ChAT-Cre/GCaMP6f mice at P5. They were then fixed and treated with antibodies against GFP and ChAT. Images were taken from the INL (**A**) and GCL (**B**), respectively. GCaMP6f signal was revealed by a GFP antibody (green, left panels in A and B), whereas SACs were labeled by a ChAT antibody (red, middle panels in **A** and **B)**. Overlay images show that almost all GFP$^+$ cells were ChAT$^+$ SACs (yellow, right panels in **A** and **B**). We occasionally observed that GFP$^+$ cells were ChAT$^-$ (arrows in **A** and **B**) and GFP$^-$ cells were ChAT$^+$ (arrowheads in **A** and **B**). The data suggest that ChAT-Cre/GCaMP6f mice can be used to real-time monitor Ca^{2+} waves of SACs in a living retina.

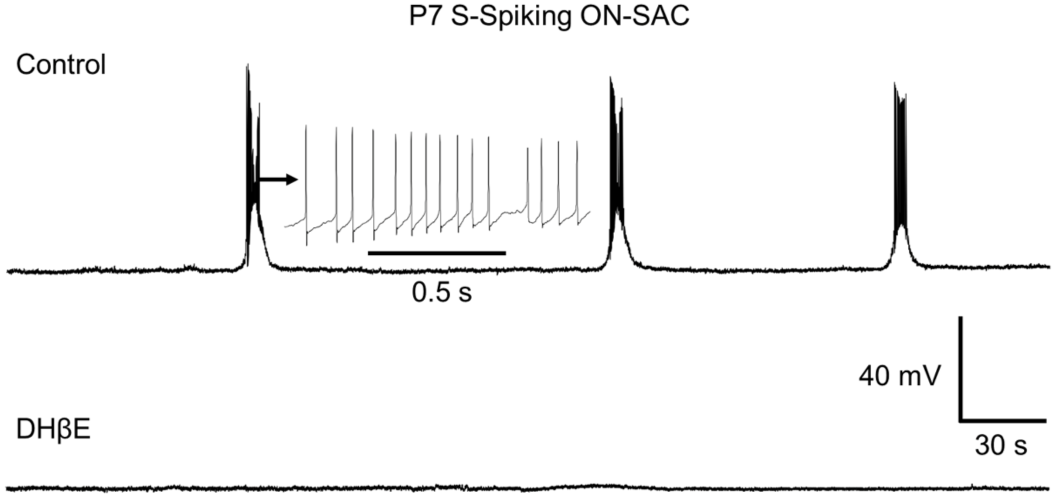

Supplementary Figure 2. ACh receptors mediate spontaneous depolarization of ON-SACs during the first postnatal week. Whole-cell current-clamp recordings were performed on ON-SACs from flat-mount retinas of ChAT-Cre/tdTomato mice at P7. An ON-SAC exhibited spontaneous rhythmic depolarization, and each of depolarization was accompanied by a burst of APs (top trace). An insert highlights the burst of APs on an extended time scale. The spontaneous depolarization and APs were completed blocked by 3 μM DhβE, an ACh receptor antagonist (bottom trace).

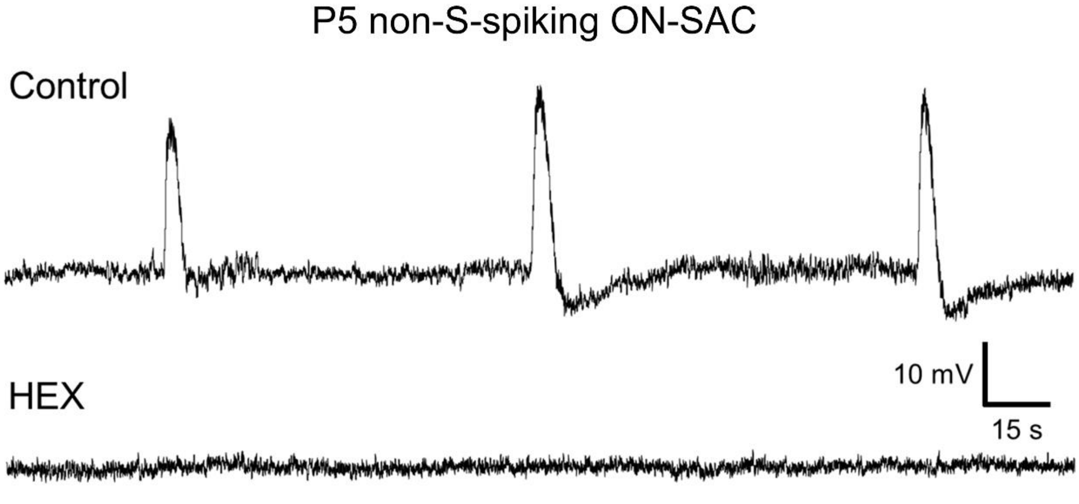

Supplementary Figure 3. ACh receptors mediate spontaneous depolarization of non-S-spiking ON-SACs during the first postnatal week. Whole-cell current-clamp recordings were performed on non-S-spiking ON-SAC from flat-mount retinas of ChAT-Cre/tdTomato mice at P5. An non-S-spiking ON-SAC exhibited spontaneous rhythmic depolarization (top trace). The spontaneous depolarization was completed blocked by 100 µM HEX, an ACh receptor antagonist (bottom trace).

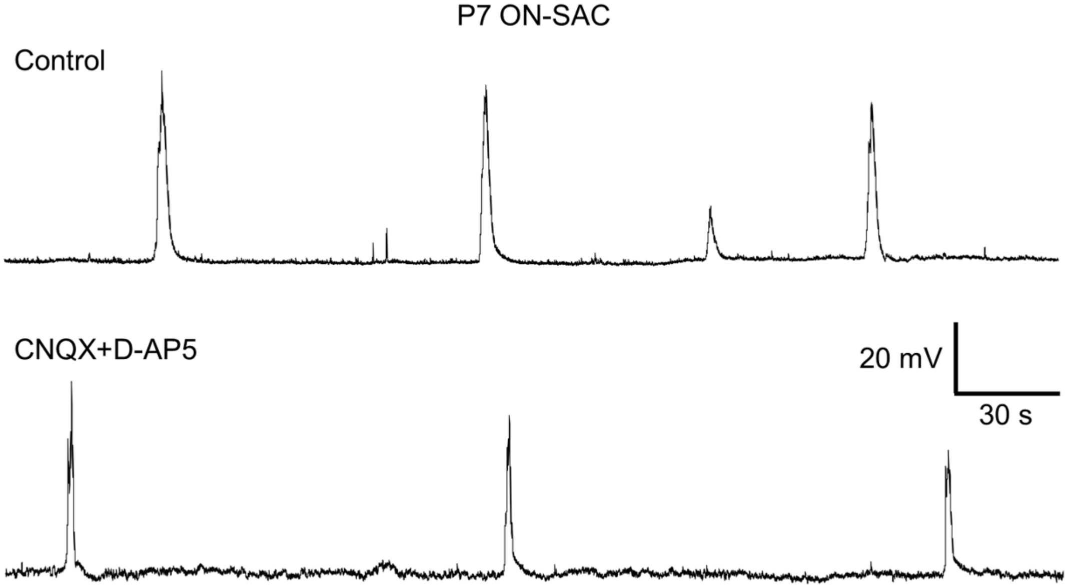

Supplementary Figure 4. Glutamate receptors are not involved in mediating spontaneous depolarization of ON-SACs during the first postnatal week. Whole-cell current-clamp recordings were performed on ON-SACs from flat-mount retinas of ChAT-Cre/tdTomato mice at P7. An ON-SAC exhibited spontaneous rhythmic depolarization (top trace). The spontaneous depolarization (bottom trace) remained unchanged in the presence of a mixture of NMDA receptor antagonist D-AP5 (50 μM) and AMPA receptor antagonist CNQX (50 μM).

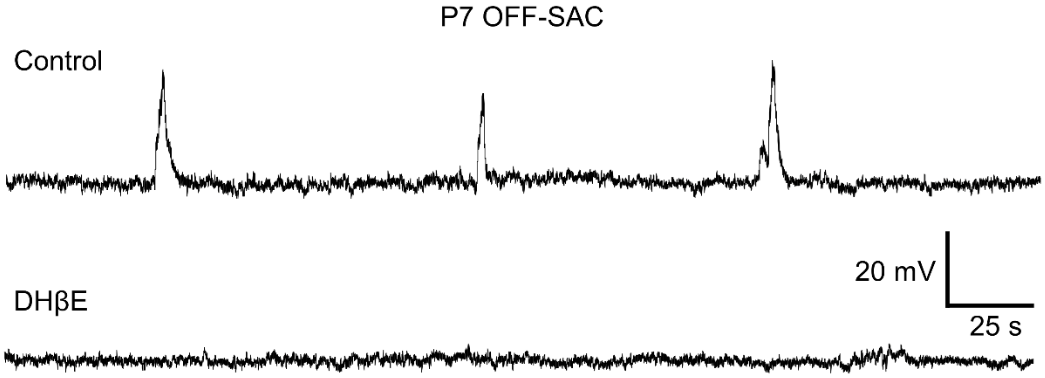

Supplementary Figure 5. ACh receptors mediate spontaneous depolarization of OFF-SACs during the first postnatal week. Whole-cell current-clamp recordings were performed on OFF-SACs from flat-mount retinas of ChAT-Cre/tdTomato mice at P7. An OFF-SAC exhibited spontaneous rhythmic depolarization (top trace). The spontaneous depolarization was completed blocked by 3 μM DhβE.

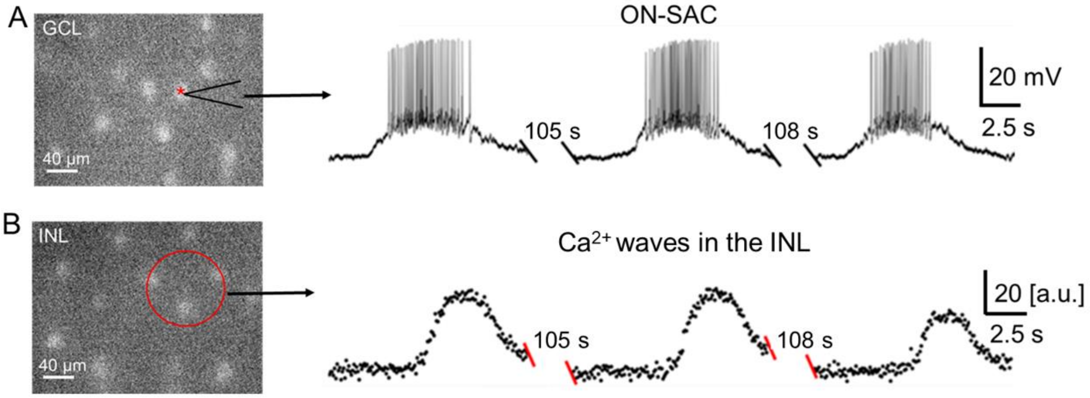

Supplementary Figure 6. Intensities of Ca^{2+} waves in the INL are correlated with the number of APs per wave in ON-SACs. Ca^{2+} imaging in the INL was simultaneously performed with whole-cell current-clamp recordings of ON-SACs in the GCL in retinas of ChAT-Cre/Ai95D mice at P5. (**A**) A live image (left panel) shows Ai95-labeled ON-SACs at P5. An arrow points to the glass pipette that was used to patch an ON-SAC. The cell exhibited spontaneous rhythmic depolarizations accompanied by APs (right panel). (**B**) Ca^{2+} images in the INL were taken simultaneously with the ON-SAC recording. A circular ROI (red, 2996.2 μm^2) of the INL located vertically above patched ON-SACs in (**A**) was used to construct a Ca^{2+} wave trace (left panel). Each Ca^{2+} wave was preceded by an electrical wave of the ON-SAC. The recording trace between each wave in **B** (right panel) was not shown (double slashes) for a period of time (indicated above the double-slashes).
